# Identifying functional relationships within sets of co-expressed genes by combining upstream regulatory motif analysis and gene expression information

**DOI:** 10.1186/1471-2164-11-S2-S8

**Published:** 2010-11-02

**Authors:** Viktor Martyanov, Robert H Gross

**Affiliations:** 1 Department of Biological Sciences, Dartmouth College, Hanover, NH 03755, USA

## Abstract

**Background:**

Existing clustering approaches for microarray data do not adequately differentiate between subsets of co-expressed genes. We devised a novel approach that integrates expression and sequence data in order to generate functionally coherent and biologically meaningful subclusters of genes. Specifically, the approach clusters co-expressed genes on the basis of similar content and distributions of predicted statistically significant sequence motifs in their upstream regions.

**Results:**

We applied our method to several sets of co-expressed genes and were able to define subsets with enrichment in particular biological processes and specific upstream regulatory motifs.

**Conclusions:**

These results show the potential of our technique for functional prediction and regulatory motif identification from microarray data.

## Background

DNA sequence motif finders are often used to predict potential regulatory motifs upstream of co-regulated genes, typically identified through gene expression experiments. The importance of upstream regulatory motifs for establishing a link between co-expression and co-regulation has been recognized previously [1-3]. These motifs represent patterns in sequence data important both for transcriptional regulation and protein function prediction [4]. However, the identification of shared motifs does not necessarily mean that the genes are involved in the same biological process. Further, microarray expression data are notoriously noisy, which impacts the ability of motif finders to identify biologically relevant patterns.

It is believed that similar gene expression profiles are the result of similar regulatory mechanisms [5]. In fact, this hypothesis served as the basis for regulatory network discovery from microarray expression experiments. However, gene expression profiles are often based on weak similarities that are unlikely to correlate with true co-regulation [6]. Potentially, there are multiple parallel regulatory mechanisms within a set of co-expressed genes. Therefore, genes displaying similar expression profiles may respond to different external stimuli, represent parallel biosynthetic pathways, and/or be regulated by different transcription factors. Thus, the problem of elucidating functional relationships and identifying potential regulatory motifs among co-expressed genes is quite challenging.

Because of the high noise level of microarray expression data, cluster analysis often returns clusters that are not functionally coherent [7]. Although the application of clustering methods to gene expression data provides numerous insights into cell regulation and disease characterization [8], the majority of current clustering algorithms do not consider functional relationships within co-expressed genes that comprise the cluster.

The majority of motif finders employ a single search strategy aimed at identifying motifs of a specific type. Because of that, they are not distinguishable from each other in terms of performance over a wide range of datasets from different species. In fact, according to the assessment of performance of thirteen different computational tools [9], absolute measures of correctness were low and similar for all the motif finders tested. It was suggested that a few tools be used in combination to improve the accuracy of predictions. This need resulted in the development of conceptually different ensemble algorithms. SCOPE (Suite for Computational Identification Of Promoter Elements), the ensemble motif finder developed in our lab [10] combines three distinct search strategies, each of which looks for a specific kind of motif: non-degenerate (e.g. ACGCGT), degenerate (ASTBKG) and long and bipartite (AYTNNNNNNNNCGT). The results of individual algorithms are then combined using a learning rule which is simply the maximum score returned by the component algorithms. SCOPE has been shown to outperform most commonly used motif finders by a statistically significant margin enjoying both high sensitivity and specificity that result in the best accuracy of transcription factor binding site prediction [10]. SCOPE is also very robust to the presence of extraneous sequences in the input gene set which makes it an excellent tool for the analysis of (often noisy) microarray data. SCOPE’s interface is also very simple and does not require the user to enter any program parameters (such as the length of the expected motif or how many instances of the motif are predicted).

**Figure 1:**
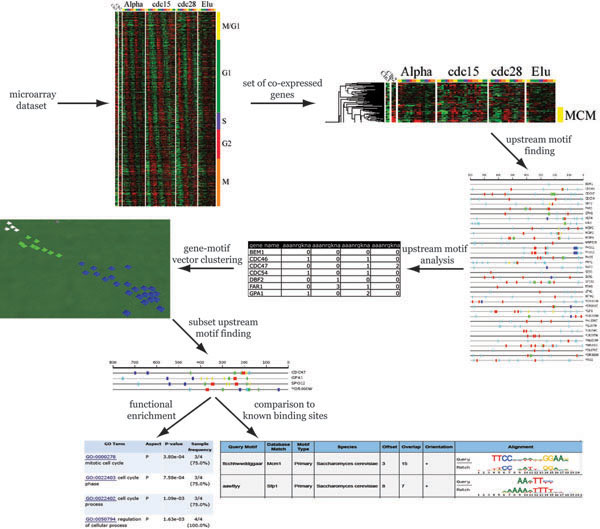
flowchart of the experimental approach.

In this paper, we describe a novel approach that examines gene expression and upstream motif data in order to generate biologically coherent subsets of genes from a starting set of co-expressed genes. Our method uses as input a set of co-expressed genes from a microarray experiment. We apply SCOPE to identify statistically significant motifs in the upstream regions of the co-expressed genes. We then convert the output of SCOPE into a motif distribution table that lists the number and positions of all occurrences of statistically significant motifs for each gene in the gene set. These data are clustered and visualized, displaying subsets of the original genes that contain similar upstream motif profiles. These new clustered gene subsets are then analyzed for functional enrichment compared to the starting gene set. Finally, statistically significant motifs found in each of the subsets are compared to the known regulatory sequences for the relevant transcription factors. Figure 1 shows overall experimental approach.

## Methods

### Sets of co-expressed genes

We used four sets of co-expressed genes from *Saccharomyces cerevisiae* microarray experiments. A set of genes upregulated during G1/S cell cycle transition was retrieved by literature mining [11,12]. We also analyzed two gene sets from a classic microarray experiment [13] that correspond to G1 CLN2 and M-G1 MCM clusters. Finally, we analyzed a set of co-expressed potential targets of the filamentous growth pathway previously identified using a rigorous statistical approach [14]. Gene expression data is clustered to generate subclusters of genes that share similar expression profiles. Each subcluster is analyzed by SCOPE to generate significant candidate regulatory motifs, which are used to generate motif profiles for each gene. A motif profile contains the number of occurrences of each motif in each upstream quartile for all significant motifs for that gene. This process generates a vector for each gene consisting of the motif occurrence profile of that gene. These gene-motif vectors are then clustered to generate subclusters of genes that have similar motif profiles. Finally, the genes in each of these last subclusters are analyzed by SCOPE and subjected to biological functional analysis.

### Gene set subclustering

The results of SCOPE can be saved as a tab-delimited text file. This file contains list of found motifs with the following information about each individual motif: consensus sequence, count (number of occurrences), Sig value (a measure of statistical significance), coverage (fraction of the input genes containing motif of interest) and a list of upstream locations of each individual instance of the consensus sequence.

For the analysis of SCOPE output, we pursued an approach that we call quartile analysis. We divide the upstream DNA region (default length 800 bps for *S. cerevisiae*) into four equal quartiles or bins, 200 bps each. The length of 800 bps for yeast seems to be the standard accepted in the community. Bins of 200 bps usually contain a high enough number of motif occurrences while simultaneously allowing one to distinguish between different motifs in terms of their distributions. Using fewer (larger) bins does not distinguish positions with enough detail and using more (smaller) bins results in many bins without any motifs. We then convert the SCOPE text file into a table where each row corresponds to a gene and each column corresponds to the number of occurrences of a motif in a given quartile across upstream regions of all genes in the gene set. Therefore, we create a series of gene-motif vectors that can be compared to each other and clustered together if motif distributions are similar for genes in question.

The actual clustering of genes based on their upstream sequence signatures and visualization of resulting subsets is done by using VxInsight. VxInsight [15] is a knowledge visualization tool that uses a force-directed placement algorithm to distribute objects (vectors of information) on a plane transforming them into an easily interpretable visual landscape. The algorithm moves similar items closer together while pushing dissimilar objects away and builds up a terrain with peaks and valleys. In our case, the peaks represent genes that have similar motif content and position distributions. The gene-motif vectors are randomly scattered on a plane and subjected to an iterative process that moves similar objects closer together and dissimilar objects further apart. This movement uses a process that is similar to simulated annealing [15]. Iterations are continued until no more movement is observed. VxInsight constructs a 3-D virtual landscape from the concentration of objects (node) on the plane. This approach does not require a pre-defined number of clusters since the data objects are not explicitly members in a particular cluster. When the process is complete, the different peaks can be selected based on the visual analysis and their content (genes) analyzed further (see Figure 1).

### Functional enrichment analysis

Gene sets generated by VxInsight were analyzed via the AmiGO [16] functional analysis tool. Subsets of the original gene set were compared in terms of either enrichment of existing functional categories or emergence of new ones.

### Transcription factor binding site search

Statistically significant motifs from SCOPE analysis of gene subsets were used as the input to search for similar motifs in the UniPROBE database [17]. This database hosts data from universal protein binding microarray assays on *in vitro* DNA binding specificities of proteins. UniPROBE enables a user to search for transcription factor binding sites in a query DNA sequence. SCOPE motifs were tested for matches with regulatory motifs in the database.

## Results

### G1/S cell cycle transition

Gene expression during G1/S transition of the cell cycle in *S. cerevisiae* is regulated by two transcription factors, MBF and SBF (Mlu1 box and Swi4/6 cell cycle box binding factor, respectively). These are heterodimeric complexes sharing a common regulatory subunit but with different DNA-binding subunits [18]. They regulate transcription of genes involved in DNA synthesis and DNA repair, budding and spindle pole body formation [19].

**Figure 2:**
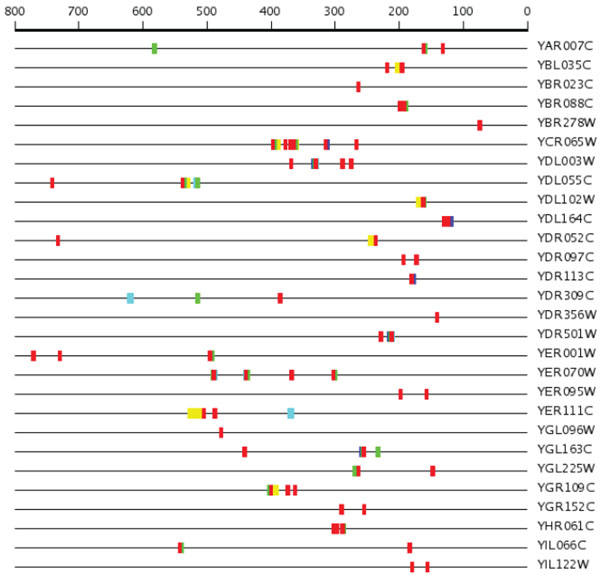
SCOPE output for some G1/S regulated genes.

Figure 2 shows a graphical SCOPE output for a set of genes upregulated during G1/S transition. The highest-scoring motif (red in the Figure 2) with the consensus sequence DWCGCGW was primarily positioned in the 100-500 bps region upstream of the gene start. See additional file 1: G1-S upregulated for complete SCOPE output.

**Figure 3:**
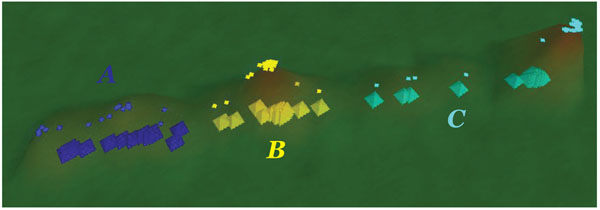
VxInsight clusters of G1/S genes.

We analyzed the SCOPE output by quartile analysis to look for gene clusters. There were three distinct VxInsight gene subsets (peaks) as shown in Figure 3. Each of the new subsets was analyzed with SCOPE and AmiGO.

**Figure 4:**
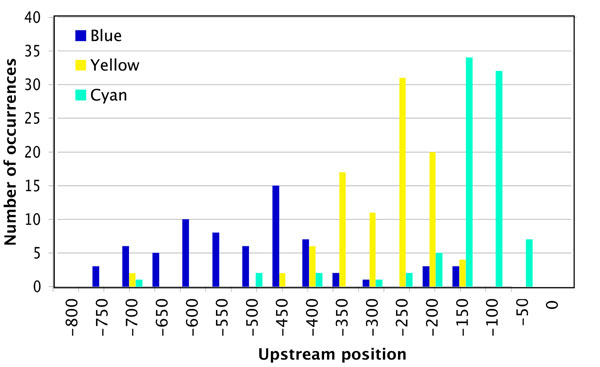
Main motif distribution in MBF-SBF clusters.

Results of the main motif distribution from the SCOPE runs are shown in Figure 4. It is interesting to point out that despite essentially sharing a CGCG core between their most statistically significant motifs (CGCGWH for blue subset, DWCGCGW for yellow subset and DNWCGCGW for cyan subset), each cluster displayed a distinct positional bias. Specifically, in cluster A (blue in Figure 3) the highest-scoring motif occurred mostly in 400-700 bps upstream, in cluster B (yellow) in the 200-400 bps upstream and in cluster C (cyan), in 100-200 bps upstream. See additional files 2, 3, 4: G1-S cluster A-C for complete SCOPE output.

These subsets were then analyzed via AmiGO to look for enrichment of functional categories within each subset. The subsets showed different functional associations. Cluster A showed significant enrichment in external encapsulating structure organization and cell wall organization (p-value ∼ 10^-6^), biological processes not found in the initial gene set analysis. Cluster C was generally similar to the main gene set in terms of overrepresented functional categories. However, it showed significantly better p-value (by 10^2^-10^4^ fold) for several biological processes including DNA repair, response to stress and cellular response to stimulus. Cluster B was very similar to main gene set and did not show any functional enrichment. Most of the genes in cluster A were known to be SBF regulated genes, while those in cluster C were MBF regulated genes. Finally, the highest-scoring motifs from the VxInsight subsets were compared to the biologically predicted regulatory sequences for SBF and MBF. Sequences from cluster A were found to be similar to the SBF transcription factor binding site (CRCGAAA) and motifs from cluster C subset were found to match closely MBF transcription factor binding site (ACGCG) [19].

### G1 CLN2 gene set

The G1 CLN2 cluster contains 76 genes, many of which are involved in DNA replication. Their expression is strongly cell cycle-regulated, with peak expression occurring in the mid-G1 phase [13].

Results of the SCOPE analysis of the G1 CLN2 show a highest-scoring motif with the consensus sequence DWCGCGW mostly located in the 100-400 bps upstream region (data not shown).

**Figure 5:**
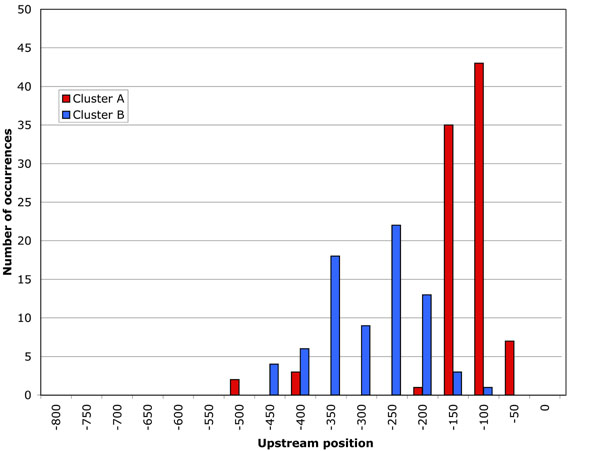
Main motif distribution in G1-CLN2 clusters.

VxInsight clustering of G1 CLN2 SCOPE output results in two gene subsets. Figure 5 shows the position distribution of the motifs in these two gene clusters. As in the SBF-MBF gene target set analysis, the two clusters showed very distinct upstream region distributions: 200-400 bps for cluster A and 100-200 bps for cluster B. See additional files 5, 6: G1-CLN2 cluster A-B for complete SCOPE output.

Functional enrichment analyses of G1 CLN2 subsets did not reveal any GO term p-value improvements over the original gene set. Generally, cluster B showed a list of functional annotations highly similar to that for the main gene set with somewhat less statistically significant p-values. Cluster A showed a much less diverse composition in the list of functional annotations with much less statistically significant p-values than the original gene set.

We then used UniPROBE to determine known motifs in each of the clusters. Different motifs were identified for the two subsets (Table 1). Statistically significant motifs from cluster A were most similar to the regulatory sequence to which Mbp1 (DNA-binding subunit of MBF) binds. Four highly-scoring SCOPE motifs from cluster B reliably matched several other UniPROBE regulatory sequences (excluding Mbp1).

**Table 1:** Comparison of G1-CLN2 subset motifs to UniPROBE data.

Gene set	SCOPE motifs	UniPROBE match	E-value	Motif coverage
G1 CLN2	dwcgcgw	Mbp1	3.0E-03	94.1%

cluster A				

				

G1 CLN2	acgcgwnd	Mbp1	2.2E-04	91.4%

cluster B	aaannnnnnnacgc	Mbp1	1.3E-03	51.4%

		Rpn4	1.6E-03	

		Fhl1	7.6E-03	

### M-G1 MCM gene set

**Figure 6:**
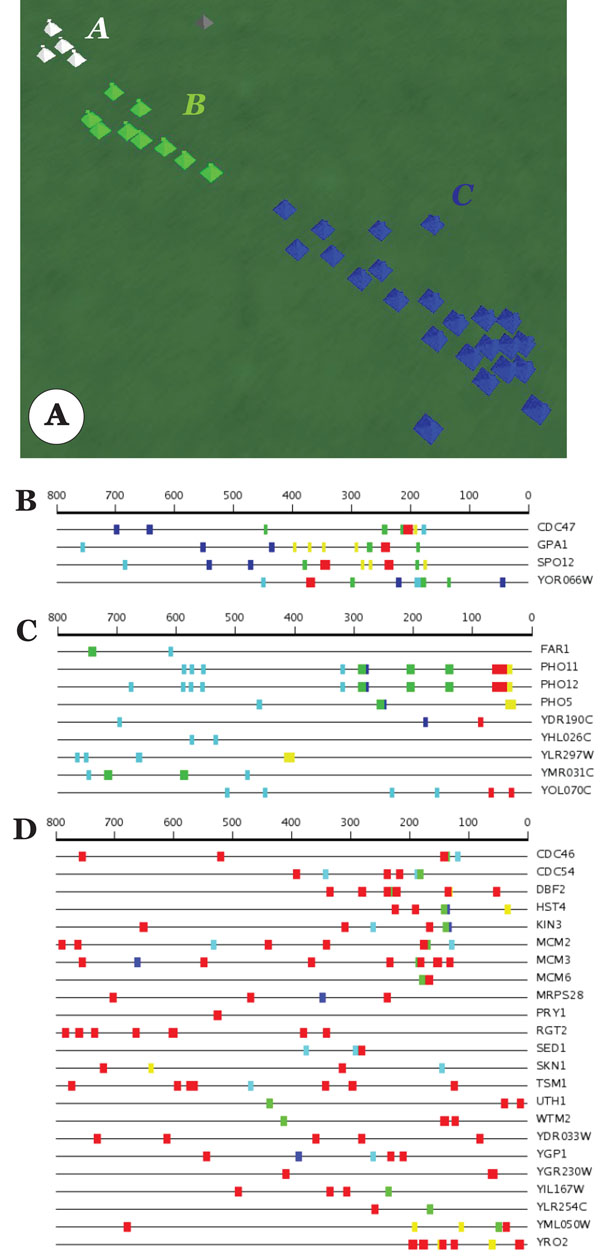
M-G1 MCM analysis. A. VxInsight clusters, B. Cluster A genes, C. Cluster B genes, D. Cluster C genes.

The M-G1 MCM cluster contains 34 genes including those that are directly involved in DNA replication. These genes peak late in the cell cycle, at about M-G1 boundary [13]. Despite numerous high-scoring motifs found in the SCOPE run, there is no easily identifiable upstream sequence pattern. Processing SCOPE output with VxInsight resulted in three subsets with visually distinct upstream motif patterns (Figure 6). See additional files 7, 8, 9: M-G1 MCM cluster A-C for complete SCOPE output.

AmiGO analysis showed enrichment in different functional terms for the three subsets. Cluster A was found to be relatively enriched in the biological processes of mitotic cell cycle, cell cycle phase and regulation of cellular process. Cluster B contained genes involved in ribosome biogenesis. Cluster C was essentially the same as the entire gene set but with less significant p-values.

Finally, in comparison to the UniPROBE database motifs, each subset displayed a characteristic upstream regulatory profile (Table 2). Cluster A motifs showed significant similarity to UniPROBE regulatory sequences for transcription factors Mcm1 and Sfp1. The four Cluster B motifs showed matches to six UniPROBE regulatory motifs (excluding Mcm1 and Sfp1). A SCOPE motif from the blue subset displayed similarity to Sfp1 regulatory sequence.

**Table 2:** Comparison of M1-MCM subset motifs to UniPROBE data.

Gene set	SCOPE motifs	UniPROBE match	E-value	Motif coverage
M-G1 MCM	ttcchhwwddggaar	Mcm1	6.5E-05	100.0%

Cluster A	aawttyy	Sfp1	3.9E-04	100.0%

				

M-G1 MCM	cacgtgdg	Cbf1	1.3E-03	44.4%

Cluster B		Pho4	4.5E-03	

	acgtg	Cbf1	4.9E-05	77.8%

		Rtg3	1.7E-03	

		Pho4	3.5E-03	

		Tye7	8.6E-03	

	cccnnnnngga	Mcm1	3.0E-03	77.8%

	aaacannnnnnnnttgc	Fkh1	1.0E-03	44.4%

		Fkh2	4.0E-03	

				

M-G1 MCM	ttttnnnnnnnnnntaa	Sfp1	1.5E-03	39.1%

Cluster C				

### Filamentous growth dataset

Filamentous growth pathway is a MAPK (mitogen-activated protein kinase) dependent pathway that regulates filamentous growth in yeast [14]. Cullen et al. [14] identified a set of its potential targets using rigorous statistical techniques. We used a set of 269 putative gene targets for our analysis.

**Figure 7:**
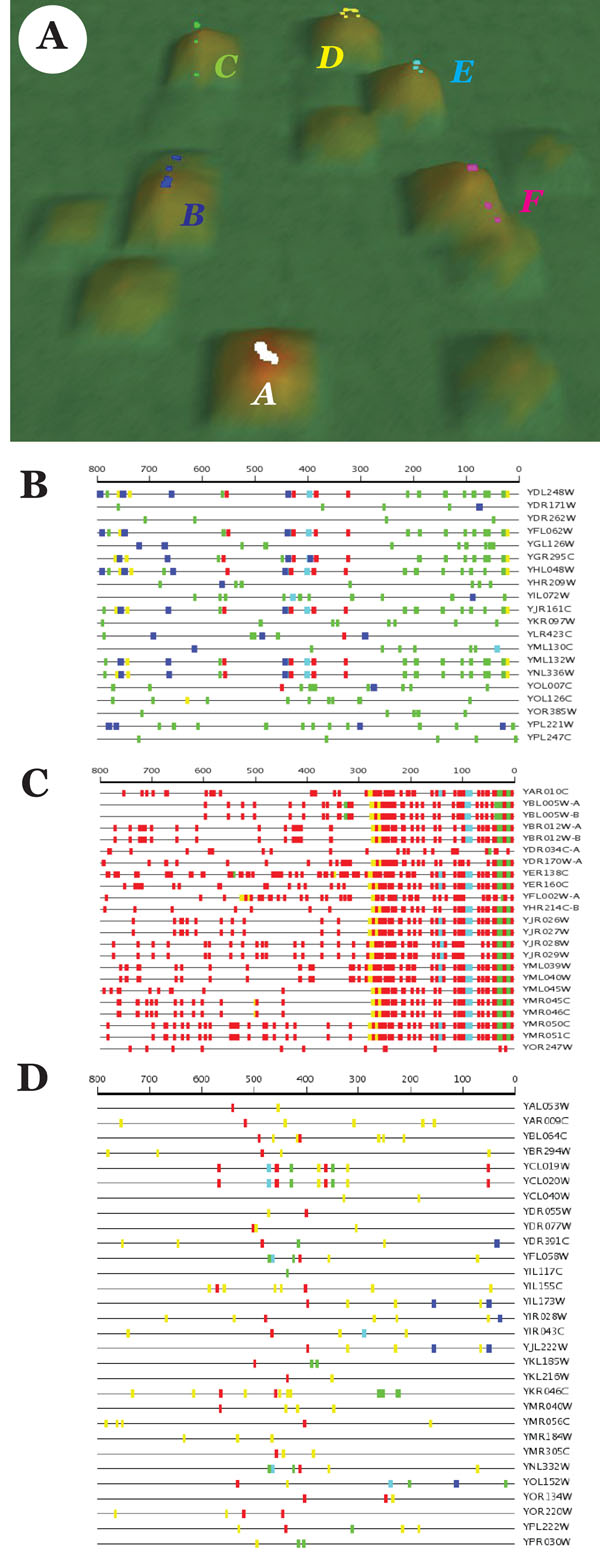
Cullen et al. dataset analysis. A. VxInsight clusters, B. Cluster C genes, C. Cluster E genes, D. Cluster F genes.

Figure 7 shows results of the VxInsight subclustering of the original gene set. We identified six distinct subsets, three of which displayed enrichment in particular biological process. Cluster A was enriched in response to temperature stimulus (p-value ∼ 10^-3^), cluster E was enriched in RNA-mediated transposition (p-value ∼ 10^-32^) and cluster F was enriched in cellular response to heat (p-value ∼ 10^-4^). In addition, cluster C was enriched in vacuole cellular component (p-value ∼ 10^-5^).

SCOPE runs of VxInsight subsets and comparisons to UniPROBE regulatory motif data showed unique upstream motif patterns in several gene sets (Table 3). There was no overlap between different subsets in terms of known regulatory motifs to which they displayed the highest similarity. See additional files 10, 11, 12, 13, 14, 15: Cullen cluster A-F for complete SCOPE output.

**Table 3:** Comparison of Cullen et al. dataset motifs to UniPROBE data.

Gene set	SCOPE motifs	UniPROBE match	E-value	Motif coverage
Cullen et al.	cataca	Rap1	7.2E-04	61.5%

Cluster B				

				

Cullen et al.	tttacannnnnnnnnnaca	Fkh1	1.2E-03	35.0%

Cluster C	cgcnnnnnnnatgt	Cup9	2.1E-03	35.0%

	atgcatga	Phd1	6.8E-03	35.0%

				

Cullen et al.	tagnnnnnnnnnatata	Spt15	1.3E-03	91.3%

Cluster E	attcc	Lys14	1.6E-03	100.0%

## Discussion

Results of the SBF-MBF gene set analysis suggest that it is possible to differentiate among transcription factor targets on the basis of sequence motif information alone. Without prior biological knowledge, we were able to associate each of the subsets with a specific regulatory pattern. Genes from cluster A are mostly regulated by SBF (15/16), genes from cluster C are mostly MBF targets (24/27) and genes in cluster B can be regulated by either or both transcription factors. It seems that in this case specific positional bias of the regulatory motif is linked to an enrichment in a particular function for the genes associated with it.

For both the G1 CLN2 and M-G1 MCM gene sets, we were able to find reliable matches with known transcriptional regulatory sequences for numerous upstream motifs predicted by SCOPE. In the case of the M-G1 MCM gene set, we identified unique functional patterns for all subsets that were generated by combining expression data and motif information.

In the case of the Cullen et al. dataset, we successfully partitioned the starting gene set into several smaller gene sets with more coherent functional annotations and unique upstream sequence signatures. Cluster E from Figure 7 is of particular interest since it displays both very significant enrichment in a particular biological process and a distinct pattern of upstream motifs across the gene set.

These results demonstrate the potential of our new approach for subdividing gene sets derived from microarray data into more functionally coherent subclusters by utilizing the upstream motif content and distribution of the genes.

## Conclusions

Motif finders have been used to identify regulatory motifs from sets of co-expressed genes determined by microarray analysis. The microarray analysis, however, is often noisy and may include several different subsets of genes that are actually regulated by different transcription factors in parallel rather than by the same transcription factor acting on all genes. By using our motif finder, SCOPE, to identify statistically significant motifs and clustering co-expressed genes based on their upstream motif content and distribution, we were able to identify subsets of genes that might represent independently regulated responses.

We applied this technique to three gene sets derived from microarray analyses and validated it by performing functional enrichment analyses and comparing computationally predicted motifs to the biologically tested regulatory sequences. We were able to generate subsets of genes with functionally enriched or novel categories and specific upstream patterns of regulatory motifs similar to known binding sites for the transcription factors. This shows the usefulness of our approach for partitioning starting gene sets into more functionally coherent subsets and making predictions about putative transcriptional regulatory patterns.

## Competing interests

The authors declare that they have no competing interests.

## Supplementary Material

Additional file 1 **G1-S upregulated.** SCOPE output for the set of genes upregulated during G1-S cell cycle transition (Figure 2). Click here for file

Additional file 2 **G1-S cluster A.** SCOPE output for the genes from G1-S cluster A (Figure 3). Click here for file

Additional file 3 **G1-S cluster B.** SCOPE output for the genes from G1-S cluster B (Figure 3). Click here for file

Additional file 4 **G1-S cluster C.** SCOPE output for the genes from G1-S cluster C (Figure 3). Click here for file

Additional file 5 **G1-CLN2 cluster A.** SCOPE output for the genes from G1-CLN2 cluster A. Click here for file

Additional file 6 **G1-CLN2 cluster B.** SCOPE output for the genes from G1-CLN2 cluster B. Click here for file

Additional file 7 **M-G1 MCM cluster A.** SCOPE output for the genes from M-G1 MCM cluster A (Figure 6). Click here for file

Additional file 8 **M-G1 MCM cluster B.** SCOPE output for the genes from M-G1 MCM cluster B (Figure 6). Click here for file

Additional file 9 **M-G1 MCM cluster C.** SCOPE output for the genes from M-G1 MCM cluster C (Figure 6). Click here for file

Additional file 10 **Cullen cluster A.** SCOPE output for the genes from Cullen cluster A (Figure 7). Click here for file

Additional file 11 **Cullen cluster B.** SCOPE output for the genes from Cullen cluster B (Figure 7). Click here for file

Additional file 12 **Cullen cluster C.** SCOPE output for the genes from Cullen cluster C (Figure 7). Click here for file

Additional file 13 **Cullen cluster D.** SCOPE output for the genes from Cullen cluster D (Figure 7). Click here for file

Additional file 14 **Cullen cluster E.** SCOPE output for the genes from Cullen cluster E (Figure 7). Click here for file

Additional file 15 **Cullen cluster F.** SCOPE output for the genes from Cullen cluster F (Figure 7). Click here for file
